# Bevacizumab alternating chemotherapy for improving the survival of patients with recurrent high-grade glioma

**DOI:** 10.1093/noajnl/vdaf157

**Published:** 2025-07-18

**Authors:** Ping-Chuan Liu, Chao-Yang Kuo, Yi-Wei Chen, Chun-Fu Lin, Shih-Chieh Lin, Feng-Chi Chang, Ming-The Chen, Jau-Ching Wu, Yi-Yen Lee

**Affiliations:** Department of Neurosurgery, Neurological Institute, Taipei Veterans General Hospital, Taipei, Taiwan; Smart Healthcare Interdisciplinary College, National Taipei University of Nursing and Health Sciences, Taipei, Taiwan; Department of Heavy Particles and Radiation Oncology, Taipei Veterans General Hospital, Taipei, Taiwan; School of Medicine, National Yang-Ming Chiao Tung University, Taipei, Taiwan; School of Medicine, National Yang-Ming Chiao Tung University, Taipei, Taiwan; Department of Neurosurgery, Neurological Institute, Taipei Veterans General Hospital, Taipei, Taiwan; Department of Pathology and Laboratory Medicine, Taipei Veterans General Hospital, Taipei, Taiwan; School of Medicine, National Yang-Ming Chiao Tung University, Taipei, Taiwan; Department of Radiology, Taipei Veterans General Hospital, Taipei, Taiwan; School of Medicine, National Yang-Ming Chiao Tung University, Taipei, Taiwan; School of Medicine, National Yang-Ming Chiao Tung University, Taipei, Taiwan; Department of Neurosurgery, Neurological Institute, Taipei Veterans General Hospital, Taipei, Taiwan; School of Medicine, National Yang-Ming Chiao Tung University, Taipei, Taiwan; Department of Neurosurgery, Neurological Institute, Taipei Veterans General Hospital, Taipei, Taiwan; Division of Pediatric Neurosurgery, Department of Neurosurgery, Neurological Institute, Taipei Veterans General Hospital, Taipei, Taiwan; School of Medicine, National Yang-Ming Chiao Tung University, Taipei, Taiwan; Department of Neurosurgery, Neurological Institute, Taipei Veterans General Hospital, Taipei, Taiwan

**Keywords:** bevacizumab alternating chemotherapy, bevacizumab, high-grade glioma, salvage treatment

## Abstract

**Background:**

High-grade glioma (HGG) is an aggressive tumor for which there are no effective therapies at recurrence, especially for isocitrate dehydrogenase (IDH)-wild-type glioblastoma. This retrospective study compared survival outcomes between patients receiving bevacizumab alternating chemotherapy (BAC) and those receiving bevacizumab (BEV) alone.

**Methods:**

We collected data from 95 adult patients with rHGG who were treated at our institute between January 2018 and August 2023. The patients were divided into 3 groups based on treatment and glioma grade: BAC regimen to treat grade 3 gliomas (*n* = 23), BAC regimen to treat grade 4 gliomas (*n* = 29), and treatment with BEV alone (*n* = 43). The BAC regimen included 2 cycles of etoposide + carboplatin, followed by 1 cycle of cyclophosphamide + vinblastine, with bevacizumab (10 mg/kg) every 4 weeks. One full cycle lasted approximately 3 months. We analyzed overall survival (OS) and postrecurrence survival (PRS).

**Results:**

In patients with grade 4 gliomas, the BAC regimen significantly improved survival compared with BEV alone, with a median OS of 29 versus 19 months and a PRS of 16 versus 10 months (both *P* < .05). In the IDH-wild-type subgroup, the BAC regimen produced a median OS of 27 versus 19 months and a PRS of 16 versus 10 months (*P* < .05). The 2-year OS and PRS rates were also higher in the BAC groups. Notably, patients with MGMT-methylated grade 4 gliomas treated with the BAC regimen had the longest median OS, 33 months.

**Conclusions:**

The BAC regimen appears effective and well tolerated in adult patients with rHGG, particularly in younger patients. Its alternating design may improve the median OS (29 vs. 19 months) and PRS (16 vs. 10 months) of patients with grade 4 gliomas while maintaining safety. As a practical option for those ineligible for clinical trials, BAC warrants further evaluation in prospective randomized studies to confirm its benefits and address the limitations of retrospective analysis.

**Key Points**

1. Bevacizumab alternating chemotherapy (BAC) may prevent resistance to a single regimen of chemotherapy and increase overall survival in patients with recurrent high-grade glioma.
2. Compared with bevacizumab alone, the BAC regimen can improve overall survival and postrecurrence survival, especially in younger patients.
3. The BAC regimen may be considered for patients who are not able to enroll in clinical trials before effective new drugs become available.

**Importance of the Study**

High-grade glioma (HGG) has been a challenging and devastating disease for several decades. While many different treatments, including surgery, radiotherapy, chemotherapy, targeted therapy, immunotherapy, and drug repurposing, are available to manage progression or recurrence, none are effective at treating recurrent HGG. Chemotherapy is the most common treatment for malignant tumors. However, prior research has indicated that chemotherapy alone is ineffective for treating rHGG. This study evaluated a bevacizumab alternating chemotherapy (BAC) regimen, which alternated between 2 combinations, etoposide (ETP) + carboplatin (CB) and cyclophosphamide (CP) + vinblastine (VBL), as an alternative to bevacizumab (BEV) monotherapy. The results indicated that patients with rHGG can benefit more from the BAC regimen than from BEV alone.

The incidence of high-grade gliomas (HGGs) such as anaplastic astrocytoma and glioblastoma (GBM) increases with age, peaking at approximately 70 years of age. GBM, the World Health Organization (WHO) grade 4 glioma with the highest incidence, has a 5-year survival rate of approximately 5%.^1–4^ Gliomas are tumors that originate from glial cells in the central nervous system. They are classified into 4 grades according to the 2007 CNS WHO histology. Additionally, the 2016 CNS WHO included molecular features to further classify gliomas.^5,6^ In the 2016 CNS WHO classification, an isocitrate dehydrogenase (IDH) gene mutation was used to subdivide GBM into 2 groups: primary GBM (IDH-wild-type) and secondary GBM (IDH-mutant). The treatment and prognosis depend on the tumor grade. According to the 2016 CNS WHO classification, the European Association of Neuro-Oncology guidelines offer further recommendations for the treatment of gliomas at diagnosis and at progression or recurrence.^7^ In IDH-mutant gliomas, treatments differ based on pathologies, such as oligodendrogliomas and astrocytomas. Prognostic factors should include patient age, neurological deficits, and the presence of residual tumors. Treatments for GBM, IDH-wild-type, and WHO grade 4 gliomas differ based on prognostic factors such as age, Karnofsky performance status (KPS), and MGMT gene mutation status. The WHO CNS5, published in 2021, offers a more precise classification for gliomas using molecular changes, IDH gene mutation status, and 1p/19q codeletion. The WHO CNS5 describes 3 types of adult-type diffuse gliomas: astrocytomas (IDH-mutant), oligodendrogliomas (IDH-mutant and 1p/19q-deleted), and GBMs (IDH-wild-type). Thus, patients can be provided with personalized treatment paths by considering histological features and molecular changes.^8^

In 2005, the Stupp protocol reported a median survival benefit of 2.5 months (14.6 vs. 12.1 months).^9^ Patients with an unmethylated 6-O-methylguanine DNA methyltransferase (MGMT) gene have longer survival due to a positive response to temozolomide.^9^ There has been no standard of care for rGBM to date; hence, numerous studies have focused on finding effective treatments.^10^ Since 2009, bevacizumab (BEV) has been used as a salvage therapy for patients with GBM. While some studies have reported prolonged survival in patients with rGBM receiving BEV alone, others have reported no significant improvement in overall survival.^11,12^ Other drugs, such as metformin, statins, NSAIDs, disulfiram, and methadone, are safe and tolerated by patients. However, their treatment outcomes are still unknown and require further clinical trials and investigations.^12^ Additionally, combining BEV with lomustine (CCNU) in rGBM also results in prolonged median progression-free survival (PFS).^11^ The benefits of combining BEV with other cytotoxic chemotherapy agents remain to be ascertained.^13^ Various immunotherapies for GBM include innate immune cell-based therapies, peptide vaccines, lymphocyte-based therapies, viral vector therapies, and nucleic acid-based therapies, which are built upon the immune escape mechanisms of glioma.^14–18^ Many active trials are underway for potential combination strategies involving immunotherapy.^19^ GBM is a refractory tumor associated with chemoresistance. Previous studies have explored alternating chemotherapy regimens in various malignancies as a strategy to improve treatment efficacy and reduce the development of drug resistance.^20–22^

In this study, we hypothesize that bevacizumab alternating chemotherapy (BAC) using 2 or more agents, as opposed to a single agent, may reduce the chance of GBM developing drug resistance. Furthermore, we propose that the BAC regimen for patients with rHGG could decrease the side effects of BEV alone and increase survival outcomes. Accordingly, in our study, we utilized a retrospective approach to evaluate the effectiveness of this novel treatment for rHGG.

## Methods

### Patient Population and Retrospective Case Review Criteria

This is a retrospective study of patients diagnosed with HGG at a single medical center, Taipei Veteran General Hospital (VGHTPE), between January 2018 and August 2023. Throughout the study period, all medical records were reviewed, and the records of 336 patients with HGGs were extracted. Participants with missing data or meeting the following criteria were excluded from the analysis: (1) initial diagnosis occurred at an age younger than 18 years or older than 70 years; (2) initial diagnosis pathology was not HGG or was unproven; (3) tumors were located in the posterior fossa, brainstem, or spine; or (4) there was no recurrence. Consequently, 95 adult patients with recurrent HGGs were initially included in the analysis. Recurrence needed to be confirmed by MRI demonstrating measurable disease according to the RANO criteria.^23^ This study received approval from the Institutional Review Board of VGHTPE (T-VGHTPE-52415) and was conducted in accordance with the guidelines of the Declaration of Helsinki.

### BAC/Bevacizumab Alone

During each admission, it was necessary to conduct blood tests and urine analyses before administering the BAC regimen or BEV alone. This study divided participants into 2 groups: those receiving the BAC regimen and those receiving BEV alone. Patients receiving the BAC regimen were further classified based on primary tumor grade: grade 3 gliomas and grade 4 gliomas.

Patients receiving the BAC regimen received a structured chemotherapy regimen consisting of 2 consecutive cycles of etoposide (ETP) + carboplatin (CB), followed by 1 cycle of cyclophosphamide (CP) + vinblastine (VBL) combined with bevacizumab (10 mg/kg) administered every 4 weeks. This 3-month sequence was defined as 1 complete BAC treatment cycle. Chemotherapy was prescribed, with ETP (80 mg/m^2^), CB (450 mg/dose), CP (800 mg/m^2^), and VBL (5 mg/m^2^) all administered intravenously. The treatment cycle included CB and ETP on day 1, followed by ETP alone on days 2 and 3. In the subsequent phase, CP was given on days 1 and 2, with VBL provided on day 3.

The BEV-alone group consisted of patients who received only BEV (10 mg/kg) every 2 weeks. Patients in all 3 groups—the BAC regimen (primary grade 3), BAC regimen (primary grade 4), and BEV-alone—continued treatment until they experienced intolerable side effects or there was evidence of disease progression.

### Statistical Analysis

The descriptive statistics of the patients were compared between the experimental and control groups. The means and standard deviations were specified for continuous variables, whereas frequencies and percentages were calculated for categorical variables. Comparisons of baseline characteristics between groups were performed using Student’s *t*-test for continuous variables and Fisher’s exact test for categorical variables. The standard for statistical significance was set at *P* < .05. Kaplan–Meier plots were produced for tumor control and OS measurements, starting from the time of rGBM treatment. In this scenario, failure events were classified as instances of mortality. Comparisons of survival distributions between groups were made using the log-rank test. All statistical analyses were performed using statistical product and service solutions and SAS 9.4 (SAS Institute).

### Data Availability Statement

The datasets generated and analyzed during this study are available from the corresponding author upon reasonable request. These data will be shared in accordance with institutional and ethical guidelines. To ensure confidentiality and maintain the integrity of the data, we will provide access through secure data transfer mechanisms. Researchers interested in accessing these data should contact Yi-Yen Lee, M.D., Ph.D., at yylee62@gmail.com.

## Results

**Figure 1.**
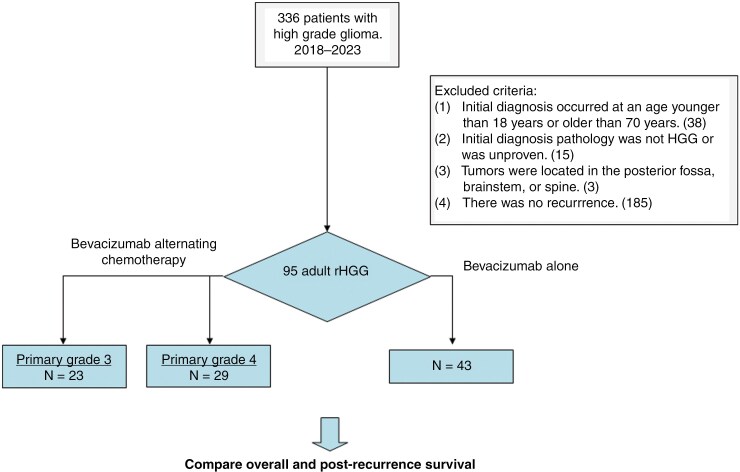
The retrospective case review criteria. The data flow diagram for the period between January 2018 and August 2023. Throughout the study duration, a comprehensive review of all medical records was conducted, resulting in the extraction of records from 336 patients diagnosed with HGGs. Participants with missing data or meeting the following criteria were excluded from the analysis: (1) initial diagnosis occurred at an age younger than 18 years or older than 70 years; (2) initial diagnosis pathology was not HGG or was unproven; (3) tumors were located in the posterior fossa, brainstem, or spine; or (4) there was no recurrence. As a result, 95 adult patients with rHGGs were initially included in the analysis.

From January 2018 to August 2023, we collected data from 336 patients with HGG at our institution. We excluded a total of 241 patients from the study, including 41 patients who were initially diagnosed either under the age of 18 years or over the age of 70 years; 15 patients who did not have confirmed HGG pathology at initial diagnosis; 3 patients with tumors located in the posterior fossa, brainstem, or spine; and 185 patients without evidence of recurrence. We subsequently performed a thorough analysis of the remaining 95 patients with rHGG, as depicted in Figure 1. The characteristics of the 95 included patients are detailed in Table 1.

**Table 1.** Demographic Information of the Study Subjects (*n* = 95)

Characteristics	Bevacizumab alternating chemotherapy				Bevacizumab alone (*n* = 43)		*P-value*
	(Primary Grade 3, *n* = 23)		(Primary Grade 4, *n* = 29)				
Continuous variable, mean, SD	Mean ± SD		Mean ± SD		Mean ± SD		
Age at diagnosed (yr) average (range)	40.00 (17–68) ± 13.47		51.76 (23–68) ± 11.59		56.07 (20–69) ± 11.22		<.0001***
Age at recurrence (yr) average (range)	42.13 (17–69) ± 13.56		52.93 (25–69) ± 11.13		56.81 (20–69) ± 11.13		<.0001***
**Categorical variable, *N*, %**	***N* (%)**		***N* (%)**		***N* (%)**		
Sex							*.3110*
Male	11	(47.83%)	16	(55.17%)	16	(37.21%)	
Female	12	(52.17%)	13	(44.83%)	27	(62.79%)	
KPS							*.8968*
90–100	2	(8.70%)	4	(13.79%)	7	(16.28%)	
70–80	4	(17.40%)	5	(17.24%)	9	(20.93%)	
<70	17	(73.90%)	20	(68.97%)	27	(62.79%)	
Primary anatomic location							*.5886*
Cortical (F-T-P-O)	14	(60.87%)	21	(72.41%)	31	(72.09%)	
Subcortical structures	9	(39.13%)	8	(27.59%)	12	(27.91%)	
Extent of surgery							<.0001***
Biopsy	5	(21.74%)	0	(0.00%)	1	(2.33%)	
Operation							
Total (GTR)	7	(30.43%)	12	(41.38%)	41	(95.34%)	
Subtotal (STR)	11	(47.83%)	17	(58.62%)	1	(2.33%)	
Primary grade							<.0001***
High-grade							
Grade3	23	(100.00%)	0	(0.00%)	0	(0.00%)	
Grade4	0	(0.00%)	29	(100.00%)	43	(100.00%)	
IDH							.0024**
Mutation	5	(21.74%)	2	(6.90%)	1	(2.33%)	
Wild type	13	(56.52%)	21	(72.41%)	41	(95.34%)	
Unknown	5	(21.74%)	6	(20.69%)	1	(2.33%)	
MGMT							.0094**
Methylated	12	(52.17%)	8	(27.59%)	27	(62.79%)	
Unmethylated	6	(26.09%)	15	(51.72%)	15	(34.88%)	
Unknown	5	(21.74%)	6	(20.69%)	1	(2.33%)	
Recurrence grade							<.0001***
High-grade							
Grade3	19	(82.60%)	0	(0.00%)	0	(0.00%)	
Grade4	4	(17.40%)	29	(100.00%)	43	(100.00%)	
Postrecurrence treatments							.0031**
Surgery	11	(50.00%)	14	(48.28%)	20	(46.51%)	
Radiotherapy	11	(50.00%)	4	(13.79%)	1	(2.33%)	

SD, Standard deviation.

### Patient Characteristics

The median age at diagnosis for the patients receiving the BAC regimen was 40.00 years (range: 18–68 years) for those with primary grade 3 gliomas and 51.76 years (range: 23–68 years) for those with primary grade 4 gliomas. This is compared to 56.07 years (range: 20–69 years) among patients receiving BEV alone. Similarly, the median age at recurrence among patients receiving the BAC regimen was 42.13 years for those with primary grade 3 gliomas and 52.93 years for those with primary grade 4 gliomas, compared to a median age was 56.81 years among patients receiving BEV alone. There were significant differences in age at diagnosis and age at recurrence among the groups (*P* < .05).

Among patients receiving the BAC regimen, those with primary grade 3 gliomas (*n* = 23) included 11 males (47.83%) and 12 females (52.17%), while those with primary grade 4 gliomas (*n* = 29) included 16 males (55.17%) and 13 females (44.83%). Patients receiving BEV alone (*n* = 43) included 16 males (37.21%) and 27 females (62.79%). No significant difference was observed in terms of sex distribution among the groups (*P* = .3110).

The KPS was assessed before the first round of chemotherapy in patients receiving the BAC regimen and before the initial administration of BEV in those receiving BEV alone.

In patients with primary grade 3 gliomas receiving the BAC regimen, 2 patients (8.70%) had a KPS of 90–100, 4 patients (17.40%) had a KPS of 70–80, and 17 patients (73.90%) had a KPS of less than 70. In patients with primary grade 4 gliomas receiving the BAC regimen, 4 patients (13.79%) had a KPS of 90–100, 5 patients (17.24%) had a KPS of 70–80, and 20 patients (68.97%) had a KPS of <70. Among patients receiving BEV alone, 27 patients (62.79%) had a KPS of 90–100, 9 patients (20.93%) had a KPS of 70–80, and 7 patients (16.28%) had a KPS of less than 70. No significant difference was observed in terms of KPS among the groups (*P* = .8968).

The primary anatomical locations of HGGs were distributed among both cortical and subcortical structures. Among patients with primary grade 3 gliomas receiving the BAC regimen, 14 patients (60.87%) had tumors located in cortical structures, whereas 9 patients (39.13%) had tumors in subcortical structures. Among patients with primary grade 4 gliomas receiving the BAC regimen, 21 patients (72.41%) had tumors located in cortical structures, whereas 8 patients (27.59%) had tumors in subcortical structures. Among patients receiving BEV alone, 31 patients (72.09%) had tumors situated in cortical structures, whereas 12 patients (27.91%) had tumors in subcortical structures. There were no significant differences in the distribution of primary anatomical locations among these groups (*P* = .5886).

The majority of patients in this study underwent surgical intervention (93.69%); only 6 patients (6.31%) underwent biopsy alone. Among patients with primary grade 3 gliomas who received the BAC regimen, 5 patients underwent biopsy, whereas 18 patients underwent surgery. This included 7 cases of gross total removal and 11 cases of subtotal removal. Among patients with primary grade 4 gliomas who received the BAC regimen, no patients underwent biopsy; all 29 patients underwent surgery, encompassing 2 cases of gross total removal and 27 cases of subtotal removal. Among patients receiving BEV alone, 1 patient underwent biopsy, and 42 patients underwent surgery, of which 41 cases were gross total removal and one case was subtotal removal. There were significant differences in the surgical approach observed among the groups (*P* < .05).

Among patients with primary grade 3 gliomas who received the BAC regimen, 23 patients were initially diagnosed with grade 3 gliomas. This included 4 cases of IDH-wild-type astrocytomas, 12 cases of anaplastic astrocytomas, 3 cases of anaplastic oligodendrogliomas, and 4 cases of anaplastic oligoastrocytomas. After recurrence, 4 patients were diagnosed with rGBM, while 19 patients continued to be classified as having grade 3 gliomas because no secondary resection was performed to confirm additional pathological alterations. All 29 patients with primary grade 4 gliomas who received the BAC regimen were initially diagnosed with GBMs, and any recurrences were considered rGBMs. Similarly, among patients receiving BEV alone, all 43 patients were initially diagnosed with HGGs, specifically GBMs, and any recurrences were classified as rGBMs. There were significant differences in the primary grade and recurrence distribution among the groups (*P* < .05).

Postrecurrence treatments, including surgery and radiosurgery, were administered during the course of systemic therapy (BAC or BEV) and not afterward. These interventions were recorded and analyzed as part of the patient’s ongoing treatment during recurrence. Among patients receiving the BAC regimen, postrecurrence treatment comprised surgery in 11 patients with primary grade 3 gliomas and radiosurgery in another 11, compared with 14 and 4 patients with primary grade 4 gliomas, respectively. Among patients receiving BEV alone, postrecurrence treatment included surgery in 20 patients and radiosurgery in one. There were significant differences in postrecurrence treatment among the groups (*P* < .05).

### Patient Molecular Characteristics

A total of 23 patients with primary grade 3 gliomas receiving the BAC regimen were included. Among them, 5 patients had IDH-mutant gliomas, 13 had IDH-wild-type gliomas, and the IDH mutation status of 5 was unknown. With respect to the MGMT promoter methylation status, 12 patients had MGMT-methylated gliomas, 6 had MGMT-unmethylated gliomas, and the MGMT methylation status of 5 was unknown.

A total of 29 patients with primary grade 4 gliomas who received the BAC regimen were included. Of these, 2 patients had IDH-mutant gliomas, 21 had IDH-wild-type gliomas, and the IDH mutation status of 6 was unknown. With respect to the MGMT promoter methylation status, 8 patients had MGMT-methylated gliomas, 15 had MGMT-unmethylated gliomas, and the MGMT methylation status of 6 was unknown.

A total of 43 patients receiving BEV alone were included. Among them, 1 patient had IDH-mutant gliomas, 41 had IDH-wild-type gliomas, and the IDH mutation status of 1 was unknown. In terms of the MGMT promoter methylation status, 27 patients had MGMT-methylated gliomas, 15 had MGMT-unmethylated gliomas, and the MGMT methylation status of one was unknown. There were significant differences in the IDH mutation status and MGMT methylation status among the groups (*P* < .05).

### Survival Analysis

The study included 95 patients: 23 patients with primary grade 3 gliomas receiving the BAC regimen, 29 with primary grade 4 gliomas receiving the BAC regimen, and 43 receiving BEV alone. A median follow-up time of 24 months (range: 3–114 months) was considered. Overall survival (OS) was defined as the time from initial diagnosis to death or last follow-up (August 2023).

**Figure 2.**
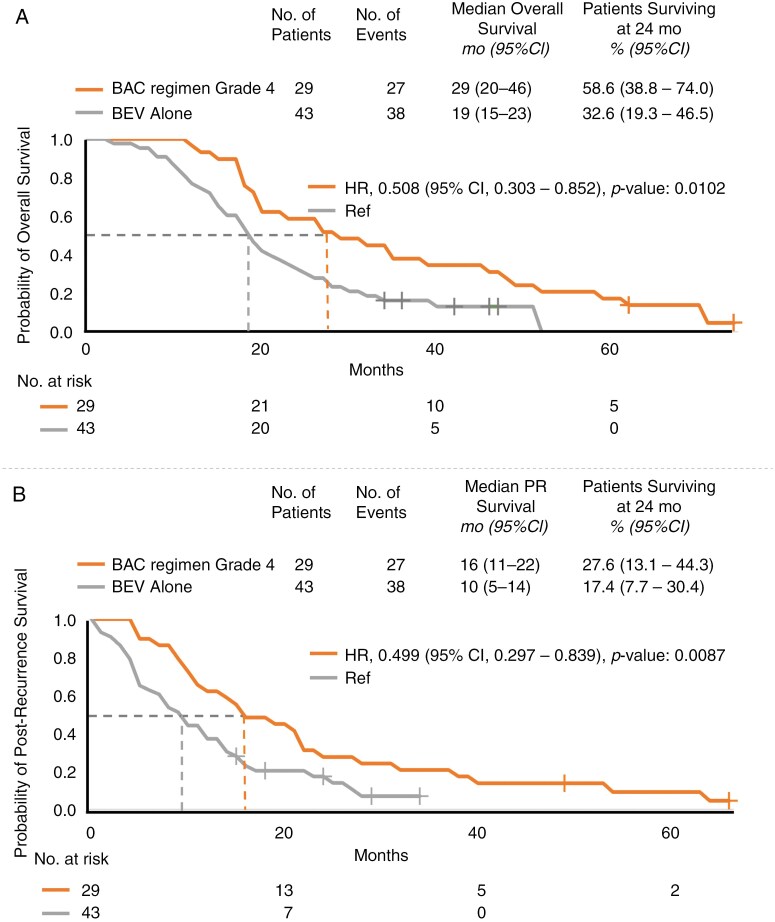
Kaplan–Meier analysis of (A) overall survival and (B) postrecurrence survival in patients with recurrent high-grade gliomas (those receiving bevacizumab alternating chemotherapy grade 4 and those receiving BEV alone). PR, postrecurrence.

In terms of the median OS, patients with primary grade 3 gliomas who received the BAC regimen had a median OS of 36 months, whereas those with primary grade 4 gliomas had a median OS of 29 months. Comparatively, patients receiving BEV alone had a median OS of 19 months (hazard ratio (HR), 0.329; 95% confidence interval [CI]: 0.179–0.602; *P* < .05; HR, 0.508; 95% CI: 0.303–0.852; *P* < .05; Figure 2A, Supplementary Figure S1A).

Regarding the median PRS, patients with primary grade 3 gliomas who received the BAC regimen had a PRS of 24 months, whereas those with primary grade 4 gliomas had a PRS of 16 months. In contrast, patients receiving BEV alone had a PRS of 10 months. The HR for the patients with primary grade 3 gliomas who received the BAC regimen was 0.358, with a 95% CI of 0.199–0.644 and a *P* value of less than .05. Similarly, the HR for the patients with primary grade 4 gliomas who received the BAC regimen was 0.499, with a 95% CI of 0.297–0.839 and a *P* value of less than .05 (Figure 2B, Supplementary Figure S1B).

Compared with patients receiving BEV alone, those receiving the BAC regimen, including patients with primary grade 3 gliomas and primary grade 4 gliomas, presented younger median ages at diagnosis (40, 51.76, and 56.07 years, respectively) and longer PFS (24, 16, and 10 months, respectively; *P* < .05). This finding indicates statistically significant differences among the groups.

At 24 months, the OS and PRS rates among patients receiving the BAC regimen were 69.6% and 46.4%, respectively, for those with primary grade 3 gliomas and 58.6% and 27.6%, respectively, for those with primary grade 4 gliomas. In contrast, among patients receiving BEV alone, the corresponding rates were 32.6% and 17.4%, respectively.

### IDH Mutation Status

There were 8 patients with IDH-mutant rHGGs: 5 with primary grade 3 gliomas and 2 with primary grade 4 gliomas who received the BAC regimen and 1 who received BEV alone. Due to the small number of patients in this subgroup, it was not feasible to conduct statistically meaningful comparative analyses.

There were 75 patients with IDH-wild-type rHGGs: 13 with primary grade 3 gliomas and 21 with primary grade 4 gliomas who received the BAC regimen and 41 who received BEV alone.

**Figure 3.**
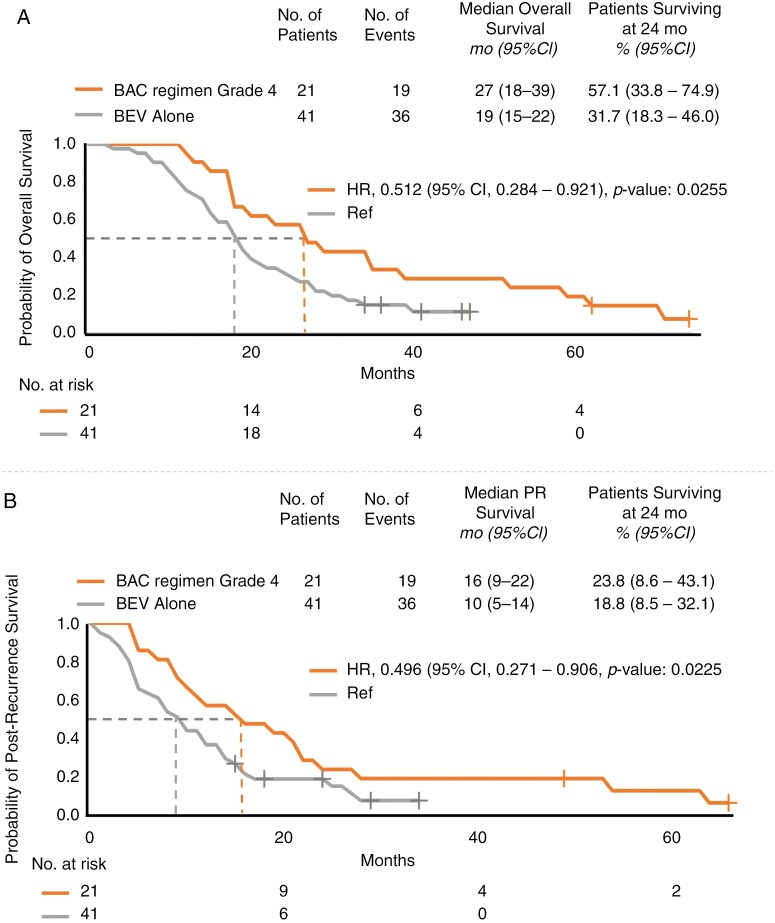
Kaplan–Meier analysis of (A) overall survival and (B) postrecurrence survival in patients with recurrent high-grade gliomas, specifically those with IDH-wild-type gliomas (those receiving bevacizumab alternating chemotherapy grade 4 and those receiving BEV alone). PR, postrecurrence.

In terms of median OS, the patients that received the BAC regimen had an OS of 28 months for those with primary grade 3 gliomas and 27 months for those with primary grade 4 gliomas. These figures contrast with the shorter 19-month OS among patients receiving BEV alone (HR, 0.478; 95% CI: 0.240–0.948; *P* < .05; HR, 0.512; 95% CI: 0.284–0.921; *P* < .05; Figure 3A; Supplementary Figure S2A). In terms of the median PRS, patients receiving the BAC regimen had a PRS of 17 months for those with primary grade 3 gliomas and 16 months for those with primary grade 4 gliomas, in contrast to the 10-month PRS observed for patients receiving BEV alone (HR, 0.543; 95% CI: 0.281–1.052; *P* = .0702; HR, 0.496; 95% CI: 0.271–0.906; *P* < .05; Figure 3B, Supplementary Figure S2B).

At 24 months, the OS and PRS rates among patients receiving the BAC regimen were 61.5% and 38.4%, respectively, for those with primary grade 3 gliomas and 57.1% and 23.8% for those with primary grade 4 gliomas. In contrast, the corresponding rates among patients receiving BEV alone were 31.7% and 18.8%, respectively.

### MGMT Methylation Status

There were 47 patients with MGMT-methylated rHGGs: 12 with primary grade 3 gliomas and 8 with primary grade 4 gliomas who received the BAC regimen and 27 who received BEV alone.

In terms of median OS, patients receiving the BAC regimen had an OS of 41 months for those with primary grade 3 gliomas and 33 months for those with primary grade 4 gliomas. This value was compared to the 21-month OS for patients receiving BEV alone (HR, 0.363; 95% CI: 0.157–0.840; *P* < .05; HR, 0.593; 95% CI: 0.250–1.406; *P* = .2353; Supplementary Figure S3A). With respect to the median PRS, patients receiving the BAC regimen had a PRS of 9 months for those with primary grade 3 gliomas and 8 months for those with primary grade 4 gliomas, compared with a PRS of 8 months for patients receiving BEV alone (HR, 0.275; 95% CI: 0.112–0.673, *P* < .05; HR, 0.655; 95% CI: 0.280–1.534; *P* = .3295; Supplementary Figure S3B).

At 24 months, the OS and PRS rates among patients receiving the BAC regimen were 75.0% and 58.3%, respectively, for those with primary grade 3 gliomas, and 62.5% and 37.5% for those with primary grade 4 gliomas. In contrast, among patients receiving BEV alone, the corresponding rates were 37.0% and 20.8%, respectively.

There were 36 patients with MGMT-unmethylated rHGGs: 6 with primary grade 3 gliomas and 15 with primary grade 4 gliomas who received the BAC regimen and 15 who received BEV alone.

With respect to the median OS, patients receiving the BAC regimen had an OS of 27 months for those with primary grade 3 gliomas and 29 months for those with primary grade 4 gliomas. In contrast, patients receiving BEV alone had a 13-month median OS (HR, 0.276; 95% CI: 0.093–0.817, *P* < .05; HR, 0.297; 95% CI: 0.132–0.670, *P* < .05; Supplementary Figure S4A). In terms of the median PRS, patients receiving the BAC regimen had a PRS of 14 months for those with primary grade 3 gliomas and 19 months for those with primary grade 4 gliomas. Meanwhile, patients receiving BEV alone presented an 8-month median PRS (HR, 0.471; 95% CI: 0.174–1.276, *P* = .1387; HR, 0.297; 95% CI: 0.129–0.683, *P* < .05; Supplementary Figure S4B).

At 24 months, the OS and PRS rates among patients receiving the BAC regimen were 50.0% and 33.3%, respectively, for those with primary grade 3 gliomas and 60.0% and 26.7% for those with primary grade 4 gliomas. In contrast, the corresponding rates among patients receiving BEV alone were 20.0% and 13.3%, respectively.

Although the sample size was small, we observed statistically significant differences in the following analyses. Administration of the BAC regimen for patients with primary grade 3 gliomas was beneficial for those with IDH-wild-type and MGMT-methylated gliomas in terms of OS (Figure 3, Supplementary Figure S4) and for those with MGMT-methylated gliomas in terms of both OS and PRS (Supplementary Figure S3). These findings were notably significant. Similarly, administration of the BAC regimen for patients with primary grade 4 gliomas was beneficial for those with IDH-wild-type and MGMT-unmethylated gliomas in terms of both OS and PRS, revealing substantial findings (Figure 3, Supplementary Figure S4).

Specifically, among patients receiving BEV alone, those with MGMT-methylated gliomas showed the longest OS, 21 months. In contrast, those with MGMT-unmethylated gliomas had the shortest OS, just 13 months. When considering PRS, patients with MGMT-methylated gliomas had a longer PRS of 12 months, while those with MGMT-unmethylated gliomas had a shorter PRS of only 8 months.

Despite the small sample size, we observed a significant difference in OS among patients with IDH-wild-type, MGMT-unmethylated rHGGs who received the BAC regimen. This effect was observed regardless of whether the gliomas were grade 3 or grade 4, compared with treatment with BEV alone (*P* < .05; Supplementary Figure S5). With respect to the PRS, we also noted a marked difference in patients with grade 3 gliomas receiving the BAC regimen compared with those receiving BEV alone. However, for patients with IDH-wild-type MGMT-methylated gliomas, no significant differences were noted in either OS or PRS (Supplementary Figure S6).

### Safety

All patients were included in the adverse event analysis (Table 2). Events are presented as those occurring in patients receiving the BAC regimen versus those receiving BEV alone; however, the groups were not statistically compared. There were no CTCAE Grade 4 or 5 adverse events. The common adverse events (Grade 1 or 2) in both groups included anemia, thrombocytopenia, neutropenia, liver enzyme impairment, renal function impairment, gastrointestinal problems, and electrolyte imbalances.

**Table 2:** Adverse Events (Number [%] of Participants Experiencing an Adverse Event, By All Group; *n* = 95)

Side effect	Grade	Bevacizumab alternating chemotherapy				Bevacizumab alone (*n* = 43)	
		(Primary grade 3, *n* = 23)		(Primary grade 4, *n* = 29)			
	Hematologic						
Anemia	Grade 1 or 2 Grade 3	15 1	(15.79%) (1.05%)	29 2	(30.53%) (2.11%)	8 0	(8.42%) (0%)
Thrombocytopenia	Grade 1 or 2 Grade 3	8 4	(8.42%) (4.21%)	16 2	(16.84%) (2.11%)	3 0	(3.15%) (0%)
Neutropenia	Grade 1 or 2 Grade 3	14 7	(14.74%) (7.37%)	19 5	(20%) (5.26%)	5 0	(5.26%) (0%)
Liver enzyme impairment AST/ALT	Grade 1 or 2 Grade 3	7 0	(7.37%) (0%)	9 1	(9.47%) (1.05%)	3 0	(3.15%) (0%)
Renal function impairment Creatine	Grade 1 or 2 Grade 3	0 0	(0%) (0%)	1 0	(1.05%) (0%)	0 0	(0%) (0%)
Blood transfusion pRBC		1	(1.05%)	8	(8.42%)	11	(11.58%)
G-CSF injection		6	(6.31%)	10	(10.53%)	3	(3.15%)
	Gastrointestinal						
Anorexia	Grade 1 or 2 Grade 3	1 0	(1.05%) (0%)	2 1	(2.11%) (1.05%)	4 0	(4.21%) (0%)
Nausea/vomiting	Grade 1 or 2 Grade 3	4 1	(4.21%) (1.05%)	1 0	(1.05%) (0%)	2 0	(2.11%) (0%)
Constipation	Grade 1 or 2 Grade 3	2 0	(2.11%) (0%)	3 0	(3.15%) (0%)	1 0	(1.05%) (0%)
Diarrhea	Grade 1 or 2 Grade 3	1 0	(1.05%) (0%)	0 0	(0%) (0%)	0 0	(0%) (0%)
Mucositis oral	Grade 1 or 2 Grade 3	3 1	(3.15%) (1.05%)	1 1	(1.05%) (1.05%)	0 0	(0%) (0%)
	Electrolytes imbalance						
Hypokalemia	Grade 1 or 2 Grade 3	4 1	(4.21%) (1.05%)	8 2	(8.42%) (2.11%)	2 0	(2.11%) (0%)
Hyperkalemia	Grade 1 or 2 Grade 3	1 0	(1.05%) (0%)	5 0	(5.26%) (0%)	0 0	(0%) (0%)
Hyponatremia	Grade 1 or 2 Grade 3	2 1	(2.11%) (1.05%)	9 3	(9.47%) (3.15%)	2 0	(2.11%) (0%)
Hypernatremia	Grade 1 or 2 Grade 3	0 0	(0%) (0%)	4 1	(4.21%) (1.05%)	0 0	(0%) (0%)
	
Alopecia	Grade 1 or 2 Grade 3	5 0	(5.26%) (0%)	2 0	(2.11%) (0%)	3 0	(3.15%) (0%)
Malaise	Grade 1 or 2 Grade 3	2 0	(2.11%) (0%)	2 0	(2.11%) (0%)	1 0	(1.05%) (0%)
Dizziness	Grade 1 or 2 Grade 3	0 0	(0%) (0%)	0 0	(0%) (0%)	1 0	(1.05%) (0%)
Infection		6	(6.31%)	13	(13.68%)	19	(20%)
Neutropenic fever		4	(4.21%)	6	(6.31%)	7	(7.37%)

Hematological adverse events were more prevalent in patients who received the BAC regimen, for those with primary grade 3 gliomas, as well as those with primary grade 4 gliomas. Among patients with primary grade 3 gliomas, neutropenia occurred in 7 patients (7.37%), and thrombocytopenia occurred in 4 patients (4.21%). Among patients with primary grade 4 gliomas, neutropenia was observed in 5 patients (5.26%), and thrombocytopenia occurred in 2 patients (2.11%). In addition to the CTCAE Grade 3 events documented in Table 2, several other Grade 3 events were reported for patients who received the BAC regimen. For patients with primary grade 3 gliomas, the reported events included anemia (1.05%), thrombocytopenia (4.21%), neutropenia (7.37%), oral mucositis (1.05%), and electrolyte imbalances such as hypokalemia (1.05%) and hyponatremia (1.09%). For patients with primary grade 4 gliomas, the reported events included anemia (2.11%), thrombocytopenia (2.11%), neutropenia (5.26%), oral mucositis (1.05%), and electrolyte imbalances such as hypokalemia (2.11%), hyponatremia (3.15%), and hypernatremia (1.05%).

Blood transfusions using packed red blood cells (pRBCs) occurred at comparable rates in both groups, including 9 patients (9.47%) receiving the BAC regimen and 11 patients (11.58%) receiving BEV alone. A combination of pancytopenia and infection-related stress necessitated pRBC transfusions for these 20 patients.

The administration of granulocyte-colony stimulating factor (G-CSF) injections was more common among patients receiving the BAC regimen than among patients receiving BEV alone. Among those receiving the BAC regimen, 6 patients (6.31%) with primary grade 3 gliomas and 10 patients (10.53%) with primary grade 4 gliomas received G-CSF injections. In contrast, only 3 patients (3.15%) among those receiving BEV alone required G-CSF injections.

Infections such as pneumonia and urinary tract infections were observed in 6 patients (6.31%) with primary grade 3 gliomas and 13 patients (13.68%) with primary grade 4 gliomas who received the BAC regimen, and 19 patients (20%) who received BEV alone. In contrast, neutropenia fever occurred more frequently among patients who received the BAC regimen group, occurring in 10 patients: 4 (4.21%) with primary grade 3 gliomas and 6 (6.31%) with primary grade 4 gliomas. These values were compared to the 7 patients (7.37%) who experienced neutropenic fever and received BEV alone.

## Discussion

Recurrent HGG is a serious disease that is difficult to treat; hence, numerous treatments and clinical trials are being conducted to discover effective strategies. Despite performing operations for resection and standard concomitant chemoradiotherapy, preventing HGG recurrence is often challenging. Patients with GBMs typically have a significantly short median survival time of approximately 15 months.^24^ BEV, which functions as a VEGF-A-targeting angiogenesis inhibitor and a modulator of tumor-induced immunosuppression, inhibits blood vessel growth and normalizes the tumor environment, thereby enhancing the effectiveness of chemotherapy.^25,26^ In patients with rHGGs, the efficacy and low toxicity of BEV have been confirmed.^27^ However, the therapeutic partners, treatment protocol, and treatment schedule of bevacizumab remain uncertain. Consequently, numerous clinical trials for rGBM are currently underway.^28^

Lomustine (CCNU), considered the main standard of care for patients with rGBM in Europe, has better survival outcomes when combined with procarbazine/vincristine, referred to as the PCV treatment, for patients with lower-grade, IDH-mutant gliomas.^29^ Current evidence indicates that the efficacy of nitrosourea alone is comparable. However, its toxicity restricts its combination with other drugs. Furthermore, a study by Wick W. et al. indicated that, compared with CCNU alone, combining BEV with CCNU could slow disease progression (HR: 0.49, *P* < .0001).^30^

As summarized in Table 3, there are studies comparing the efficacy of various chemotherapy regimens for the treatment of rHGG. The PRS observed in our BEV-alone group (10 months, *n* = 43) was comparable to that reported by Friedman et al.^31^ (9.2 months, *n* = 85), which may reflect differences in sample size and study design. Notably, the BAC regimen in our study resulted in even greater PRS than BEV-alone and outperformed previously reported combinations of BEV with CCNU, irinotecan, or carboplatin (Table 3).^30–33^ In our study, the BAC regimen may have helped mitigate tumor drug resistance and potentially improved PRS. Second-line chemotherapy with PCV or bevacizumab/irinotecan (BI) has been compared and reported by Bruno F. Carvalho.^33^ Compared with PCV, BI results in a better median OS and PRS, at 9 versus 5 months and 5 versus 3 months, respectively. In our study, the BAC regimen, administered to patients with primary grade 3 gliomas as well as those with primary grade 4 gliomas, resulted in significantly better median OS and PRS than did BEV alone. The median OS was 36 and 29 months for patients receiving the BAC regimen versus 19 months for those receiving BEV alone, whereas the median PRS was 24 and 16 months versus 10 months, respectively. The administration of BI resulted in less toxicity, with CTCAE Grades 3 and 4 (22%). As a therapeutic option, BI was superior to PCV in terms of efficacy and presented less toxicity. BEV administered both alone and in combination with other agents (such as carboplatin, vorinostat, or dasatinib) has been evaluated in randomized trials. The findings suggest that these combinations offer comparable efficacy in treating rHGG.^32^

**Table 3.** Literature Review and Comparison of the Efficacy of Various Chemotherapy Regimens in rHGG

Treatment	Postrecurrence survival (months)	*n*	Authors	Year
BEV alone	9.2	85	Friedman HS et al.^31^	2009
BEV alone	7.5	62	Field KM et al.^32^	2015
BEV alone	10	43	this study	
BEV + CCNU	9.1	288	Wick W et al.^30^	2017
BEV + Irinotecan	9	41	Carvalho BF et al.^33^	2015
BEV + Carboplatin	6.9	60	Field KM et al.^32^	2015
BAC grade 3	24	23	this study	
BAC grade 4	16	29	this study	

(Postrecurrence survival comparisons presented in this table are illustrated in Supplementary Figure S1B).

BAC, bevacizumab alternating chemotherapy; BEV, bevacizumab; CCNU, lomustine; rHGG, recurrence high-grade glioma.

Field et al.^32^ reported that carboplatin (CB), which achieves an area under the concentration-time curve (AUC) of 5 every 4 weeks, was associated with poorer OS when combined with BEV compared with BEV alone (6.9 vs. 7.5 months, respectively). Although there were no significant differences in adverse events between the groups, common events included fatigue, neurological symptoms, hypertension, nausea/vomiting, and thrombocytopenia. The group treated with both CB and BEV experienced more CTCAE Grade 3 events, such as hypertension (17%), deep vein thrombosis (3%), pulmonary embolus (3%), and gastrointestinal perforation (2%).^32^ The administration of CB as a single agent resulted in greater toxicity than did that of BEV alone, with no improvement in OS. Patients with rGBM treated with BEV in combination with VBL and low-dose CB (300 mg/m^2^) had longer OS than did patients not receiving chemotherapy (13.5 vs. 3.2 months, respectively); OS was also longer in patients younger than 50 years, as reported by Yu-Kai Huang et al.^34^

The combination of ETP and CB for patients with rHGGs has been reported, with median OS and PFS of 3.3–9 months and 3–4 months, respectively. CTCAE Grade 3 and 4 hematotoxicity occurred in 26% of patients, with 67% experiencing ototoxicity of the same grade. The occurrence of ototoxicity was observed in 4.3% of patients.^35,36^ Six patients with rGBM underwent combined chemotherapy of ETP and CB plus BEV. After 2 cycles of treatment, 5 patients achieved a partial response, and one patient developed extensive necrosis. The rates of OS and median PFS were 29.9 and 19 weeks, respectively.^37^ However, these findings from the 6-patient cohort study suggest a potential therapeutic option of combined chemotherapy plus BEV. Enrico Franceschi1 et al.^38^ reported that patients with rGBM receiving BEV as third-line therapy had better survival if they had MGMT-methylated gliomas. Compared with chemotherapy, the OS and PFS for patients receiving BEV third-line therapy were significantly different: 8.0 versus 6.0 months (*P* = .014) and 4.7 versus 2.6 months (*P* = .02), respectively. In contrast, Hovey, E. J. et al.^39^ reported that there was no significant improvement in survival rates for patients with rGBMs who continued BEV beyond progression. There are more reports about the combination of BEV with chemotherapy. A meta-analysis was reported by Shou-Bo Yang et al.,^40^ in which studies were included and analyzed.^34,41^ The chemotherapy included CB and CCNU. Therefore, future clinical trials and studies could investigate the combination of BEV with CB and CCNU. Compared with BEV or chemotherapy alone, the combination of BEV with chemotherapy improved PFS (HR, 0.66; 95% CI: *P* < .00001), whereas OS was not significantly different (HR 0.99; *P* = .92). Moreover, the concept of alternating chemotherapy has also been applied for small cell lung cancer (SCLC). K. Havemann et al. demonstrated that alternating chemotherapy combinations resulted in improved response rates and survival outcomes compared with sequential administration.^21^

Mechanistically, the BAC regimen includes etoposide, carboplatin, cyclophosphamide, and vinblastine—agents that primarily induce DNA strand breaks or disrupt microtubule function rather than causing O6-methylguanine lesions typically repaired by MGMT. This may help explain why the MGMT promoter methylation status did not consistently significantly influence OS or PRS between patients receiving the BAC regimen and those receiving BEV alone (Supplementary Figures S3 and S4). Our analysis revealed no statistically significant differences in survival outcomes between patients with MGMT-methylated gliomas and those with MGMT-unmethylated gliomas who received the BAC regimen. However, these findings are based on a limited sample size and should be interpreted with caution. Further validation in larger, prospective studies is needed.

Past studies have explored alternating chemotherapy for the treatment of other cancers.^20–22,42^ Stewart et al.^22^ found that alternating cisplatin-based regimens in patients with SCLC was feasible, with outcomes comparable to those of standard approaches. Similarly, Budd et al.^42^ reported a 54% response rate when non-cross-resistant regimens were alternated in patients with metastatic breast cancer, suggesting potential benefits in overcoming drug resistance. In our results, some patients still had significant survival benefits from the BAC regimen. According to previous studies,^20–22,42^ the use of the same chemotherapeutic agents repeatedly without switching to different drugs is ineffective. A key advantage of the BAC regimen is its alternating chemotherapy design. By rotating non-cross-resistant agents, this approach may delay the emergence of tumor resistance by altering the selective pressure on tumor cells. Each drug also has a cumulative toxicity threshold; alternating agents allow for effective multidrug treatment without exceeding the dose limits of any single agent. This may explain the favorable tolerability and improved survival outcomes observed in our cohort. For patients with rHGGs—for whom resistance is common and treatment options are limited—this strategy could offer a clinical advantage. Although we did not assess molecular markers of resistance directly, the observed efficacy and low incidence of severe adverse events support the potential of this approach. However, further studies are needed to validate this hypothesis. By alternating different chemotherapeutic regimens, we were able to reduce the likelihood of developing drug resistance in patients with rHGGs, thereby extending patient survival.

In such a devastating disease, it is crucial to consider the drug safety and side effects of BEV and chemotherapy. Therefore, evaluation of health-related quality of life also becomes an essential. Kathryn M Field et al.^43^ presented a randomized phase 2 study comparing health-related quality of life in patients with GBMs treated with BEV alone with that in patients treated with BEV plus CB chemotherapy. There were no significant differences in health-related quality-of-life outcomes between these groups. Notably, health-related quality of life will be an essential measure of OS and PRS in future clinical studies and for the treatment of patients with rGBMs.

The limitations of this study include its retrospective design, small sample size, and single-center data. As this was not a randomized controlled trial, selection bias may have influenced group allocation and outcomes. Additionally, the potential confounding effects of postrecurrence treatments—such as surgery and radiosurgery administered during systemic therapy—cannot be fully excluded. While these interventions were applied during the BAC or BEV treatment courses, their independent impact on survival outcomes remains difficult to isolate.

In conclusion, therapeutic options for adult patients with rHGGs continue to be controversial and are desperately needed. Numerous clinical trials are ongoing to determine the most effective and safe treatment for these patients. Future directions may include considering the BAC regimen and the development of new drugs.

## Conclusions

We discovered that treatment with the BAC regimen is effective and offers tolerable toxicity in adult patients with rHGGs. Compared with the administration of BEV alone, the BAC regimen improved OS and PRS. For younger patients, BAC remains a good choice. Further randomized clinical trials are warranted to confirm these results.

## Limitations

This study is a retrospective, real-world cohort with a small sample size from a single center. This was not a randomized controlled trial, and selection bias may have been present in the BAC regimen group.

## Supplementary Material

vdaf157_suppl_Supplementary_Figures_S1

vdaf157_suppl_Supplementary_Figures_S2

vdaf157_suppl_Supplementary_Figures_S3

vdaf157_suppl_Supplementary_Figures_S4

vdaf157_suppl_Supplementary_Figures_S5

vdaf157_suppl_Supplementary_Figures_S6
