# Supplementary figures and images for: Bevacizumab alternating chemotherapy for improving the survival of patients with recurrent high-grade glioma
Source: Neurooncol Adv. 2025 Jul 18;7(1):vdaf157. doi: 10.1093/noajnl/vdaf157 (PMC12311935; doi:10.1093/noajnl/vdaf157)

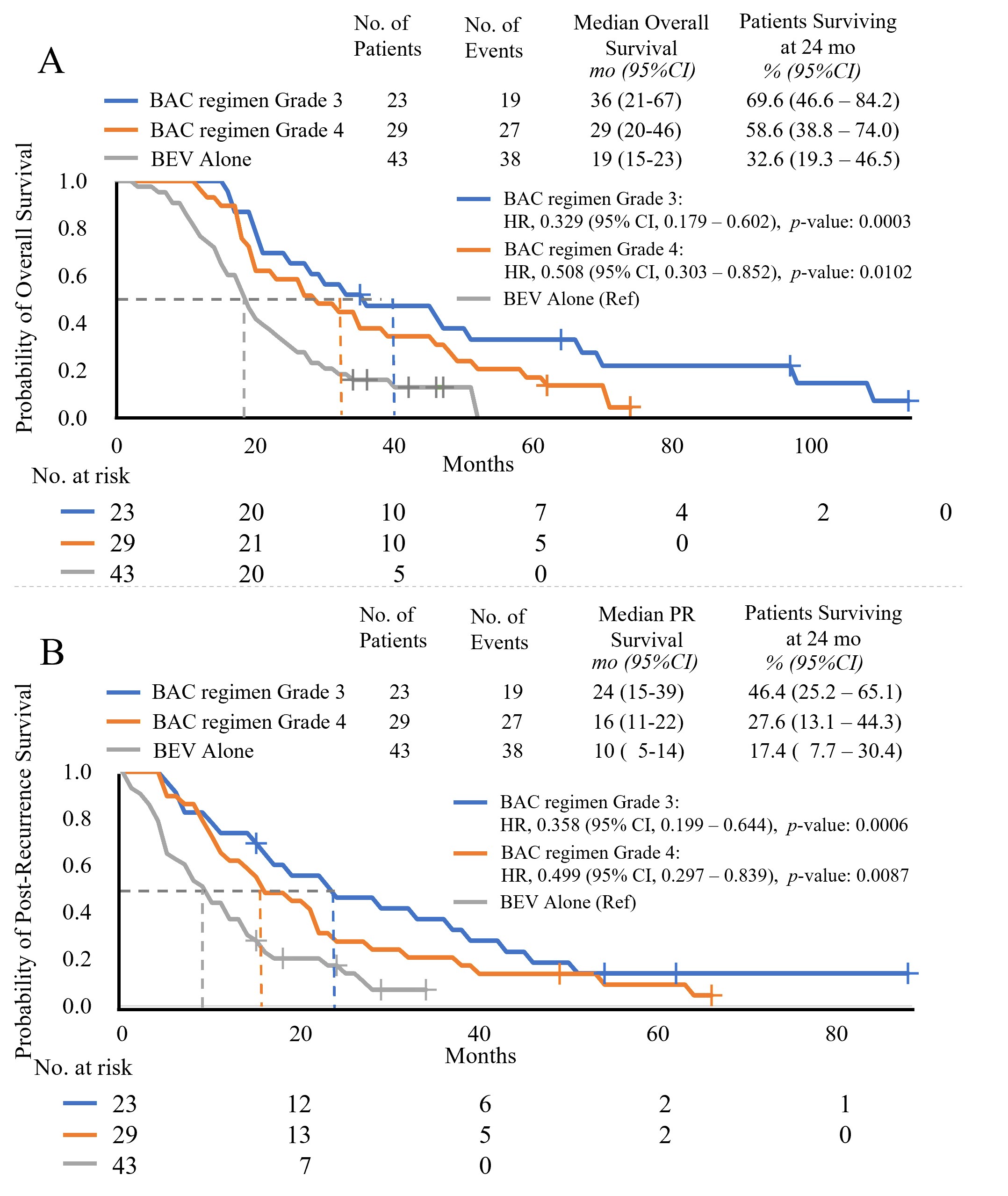

Supplement: vdaf157_suppl_Supplementary_Figures_S1 [file vdaf157_suppl_supplementary_figures_s1.jpeg]

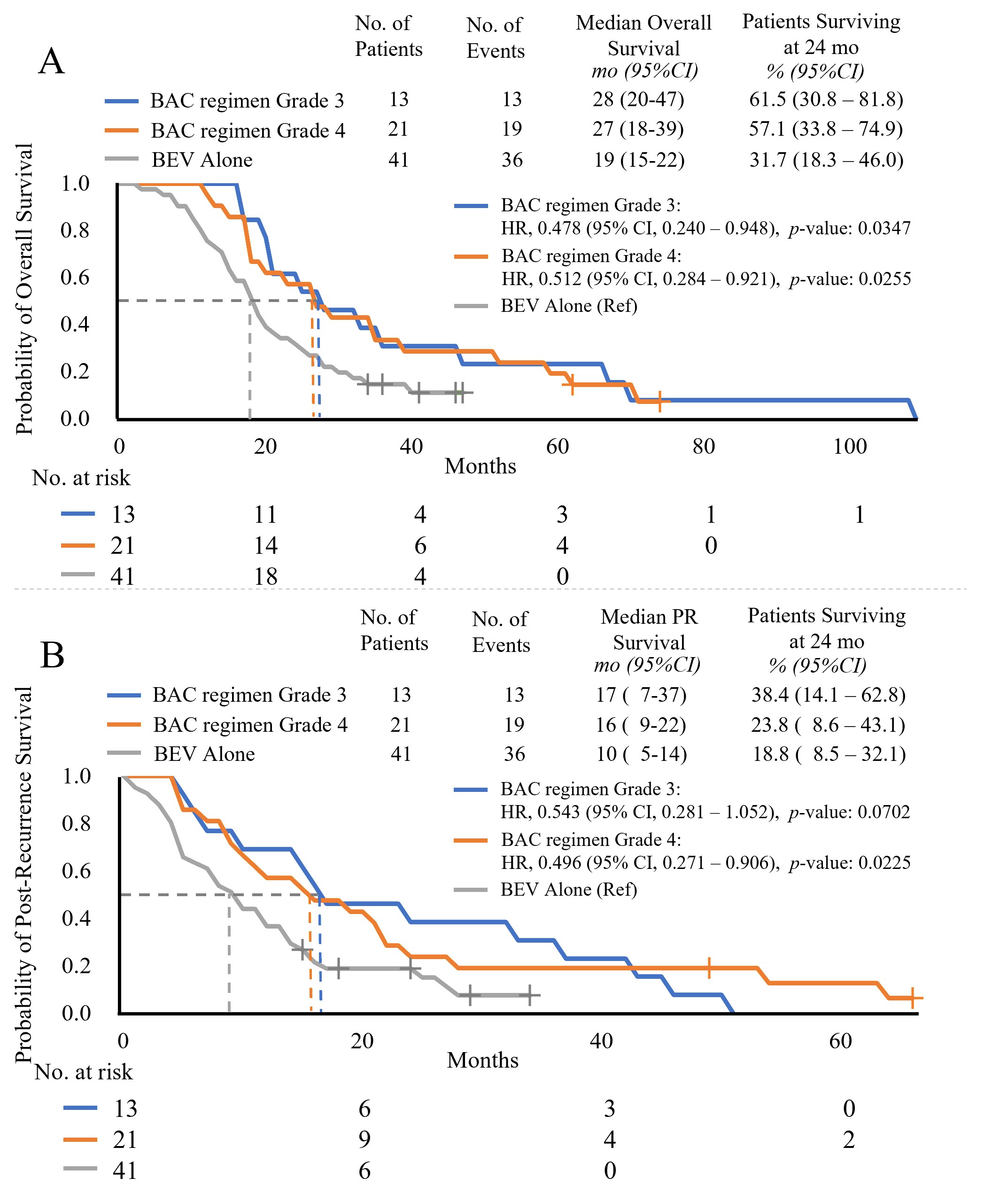

Supplement: vdaf157_suppl_Supplementary_Figures_S2 [file vdaf157_suppl_supplementary_figures_s2.jpeg]

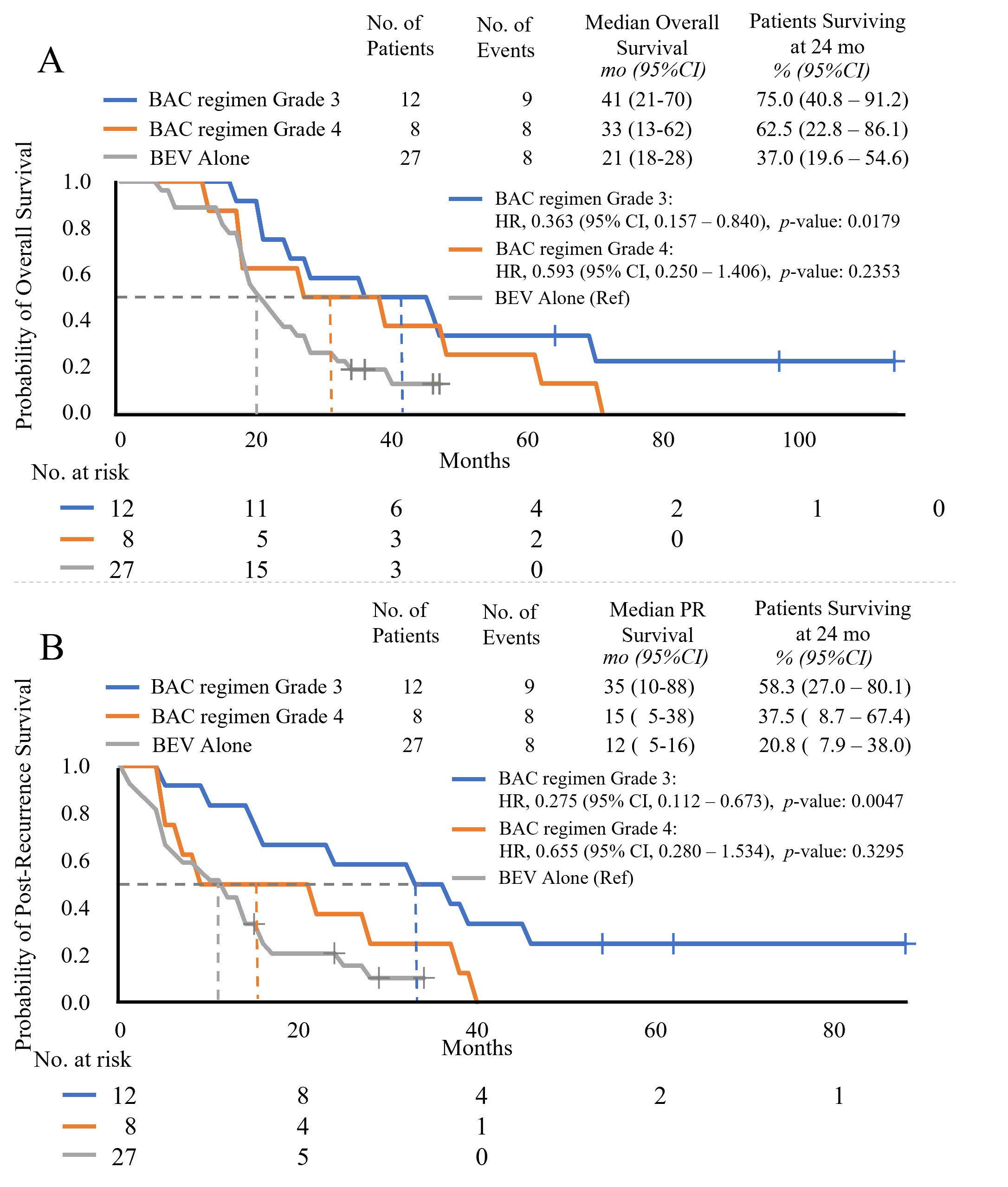

Supplement: vdaf157_suppl_Supplementary_Figures_S3 [file vdaf157_suppl_supplementary_figures_s3.jpeg]

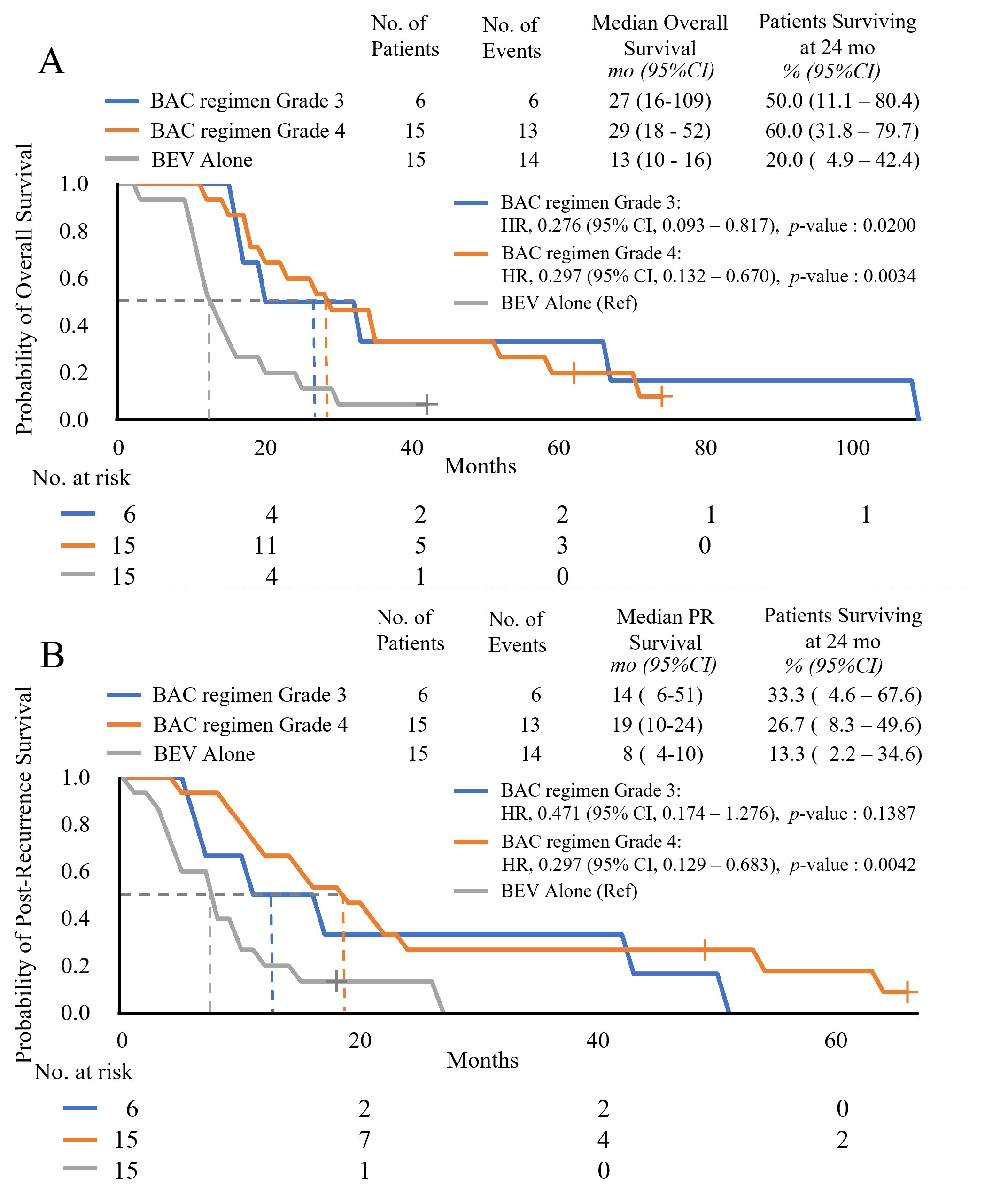

Supplement: vdaf157_suppl_Supplementary_Figures_S4 [file vdaf157_suppl_supplementary_figures_s4.jpeg]

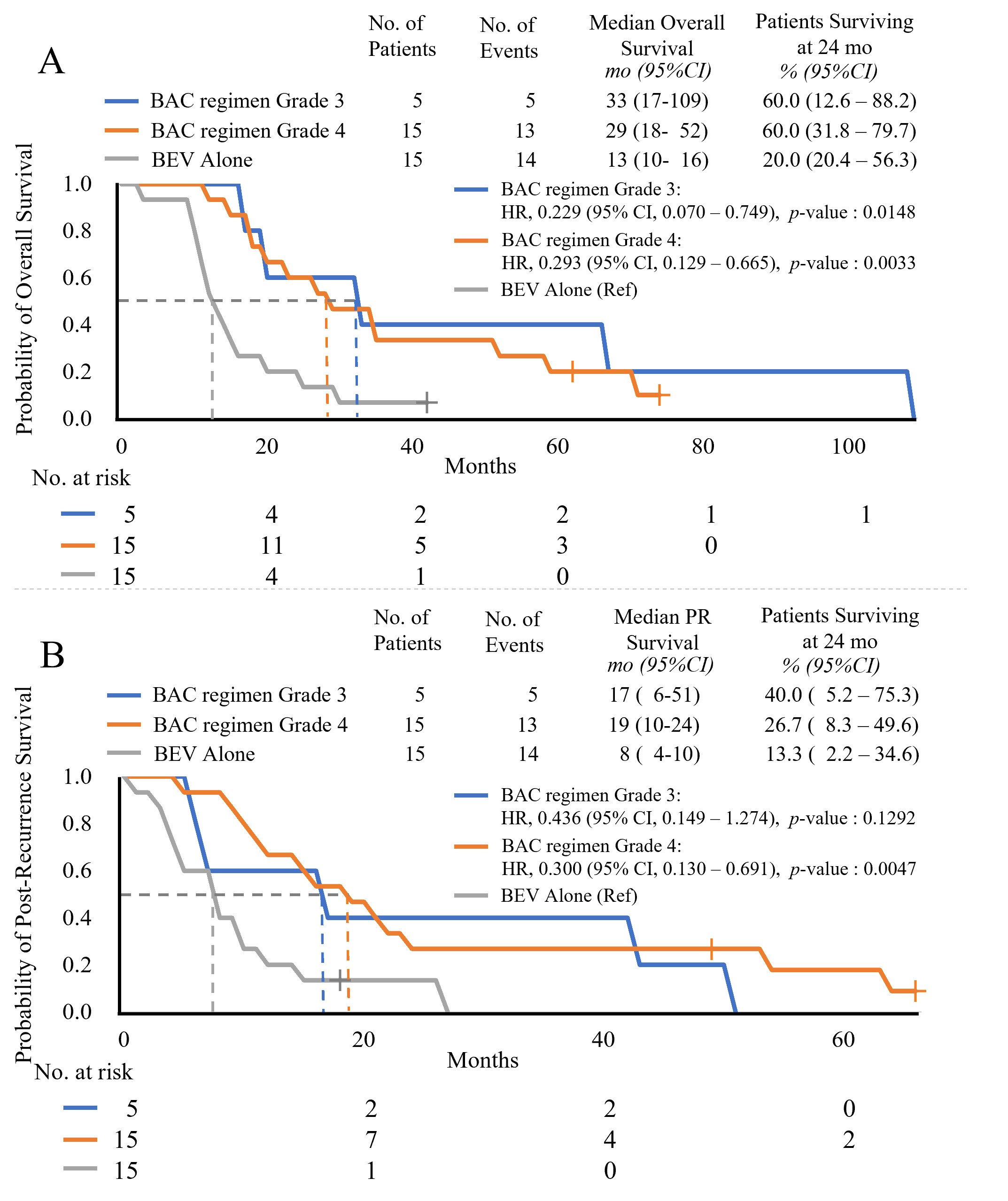

Supplement: vdaf157_suppl_Supplementary_Figures_S5 [file vdaf157_suppl_supplementary_figures_s5.jpeg]

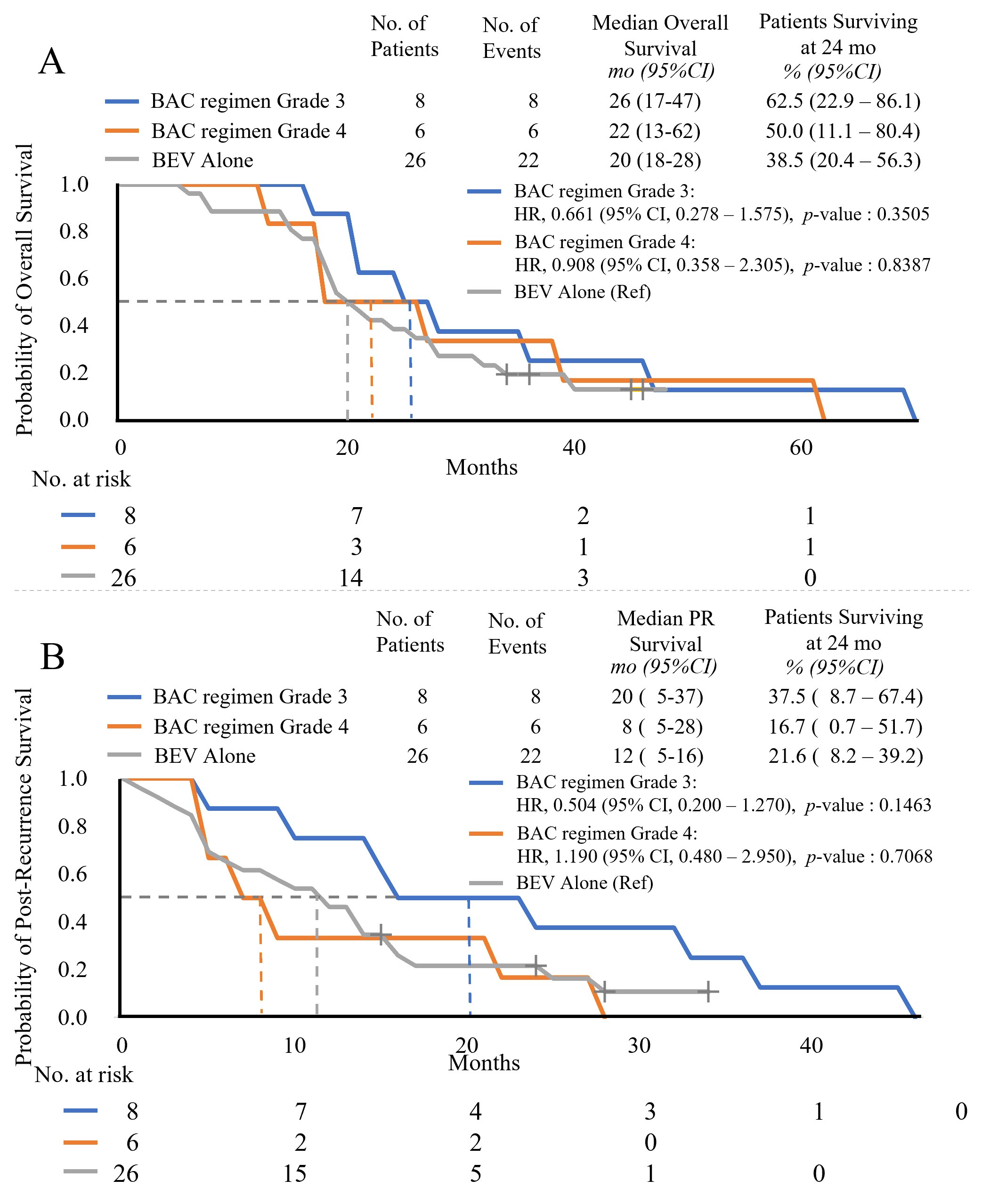

Supplement: vdaf157_suppl_Supplementary_Figures_S6 [file vdaf157_suppl_supplementary_figures_s6.jpeg]
